# Influence of SiO_2_ shell thickness on power conversion efficiency in plasmonic polymer solar cells with Au nanorod@SiO_2_ core-shell structures

**DOI:** 10.1038/srep25036

**Published:** 2016-04-29

**Authors:** Ran Zhang, Yongfang Zhou, Ling Peng, Xue Li, Shufen Chen, Xiaomiao Feng, Yuqiao Guan, Wei Huang

**Affiliations:** 1 Key Laboratory for Organic Electronics and Information Displays & Institute of Advanced Materials (IAM), Jiangsu National Synergetic Innovation Center for Advanced Materials (SICAM), Nanjing University of Posts & Telecommunications (NUPT), Nanjing 210023, China; 2 Mechanical Engineering Institute, Nanjing Institute of Technology, Nanjing 211167, China; 3 Key Laboratory of Flexible Electronics (KLOFE) & Institute of Advanced Materials (IAM), Jiangsu National Synergetic Innovation Center for Advanced Materials (SICAM), Nanjing Tech University, 30 South Puzhu Road, Nanjing 211816, China

## Abstract

Locating core-shell metal nanoparticles into a photoactive layer or at the interface of photoactive layer/hole extraction layer is beneficial for fully employing surface plasmon energy, thus enhancing power conversion efficiency (PCE) in plasmonic organic photovoltaic devices (OPVs). Herein, we first investigated the influence of silica shell thickness in Au nanorods (NRs)@SiO_2_ core-shell structures on OPV performances by inserting them into poly(3,4-ethylenedioxythiophene):poly(4-styrenesulfonate) and thieno[3,4-b]thiophene/benzodithiophene (PTB7) interface, and amazedly found that a 2–3 nm silica shell onto Au NRs induces a highest short-circuit current density of 21.2 mA cm^−2^ and PCE of 9.55%. This is primarily due to an extremely strong local field and a much slower attenuation of localized surface plasmon resonance around ultrathin silica-coated Au NRs, with which the field intensity remains a high value in the active layer, thus sufficiently improves the absorption of PTB7. Our work provides a clear design concept on precise control of the shell of metal nanoparticles to realize high performances in plasmonic OPVs.

Organic photovoltaic devices (OPVs) have attracted a considerable attention because of their advantages of light-weight, low-cost, large-scale manufacturing process and mechanical flexibility^1^,^2^. Unfortunately, in order to achieve efficient carrier extraction, the photoactive layer in OPVs must be rather thin (100 nm or less) due to their extremely low carrier mobilities for most of organic/polymer materials (on the order of 10^−4^ cm^2^/V·s). Such thin photoactive layers lead to a significant loss of incident sunlight, thereby a final low light absorption efficiency and power conversion efficiency (PCE). Recently, surface plasmons (SPs) have been widely employed in OPVs to significantly improve the light harvesting in photoactive layers and hence enhance short-circuit current density (*J*_sc_) and PCEs via amplifying local field and scattering incident sunlight^3^,^4^.

Both periodic/aperiodic metal nanoarrays^5^,^6^,^7^ and discrete metal nanoparticles^8^,^9^,^10^,^11^,^12^,^13^,^14^,^15^ were used to generate SPs, and the latter was widely reported due to ease fabrication with a variety of shapes (e.g., nanospheres^8^,^9^, nanoprisms^10^, nanorods (NRs)^11^,^12^,^13^ and nanocubes)^14^,^15^. Among all kinds of nanoparticles, both Au nanocubes and Au NRs generate a much stronger localized surface plasmon resonance (LSPR) than the other shapes. But very unfortunately, the local field around Au nanocubes declines very quickly (Figure S1 in Supplementary Information), and doping them into a carrier extraction layer leads to an extremely limited increase in PCE (e.g., only 9.3%)^14^. In contrast to Au nanocubes, Au NRs show a much slower decline in the local field intensity and their adjustable longitudinal/transverse ratios make them be widely applied in OPVs with both broad and narrow band-gap active materials^16^.

Although generating a magnified local field, these metal nanoparticles also act as recombination centers of excitons, thus they are often located into a functional layer (e.g., the poly(3,4-ethylenedioxythiophene):poly(4-styrenesulfonate) (PEDOT:PSS) film) adjacent to the photoactive layer to avoid direct contacts with excitons. As a result, nanoparticles-induced local field amplification and light scattering cannot be totally absorbed by the photoactive layer, resulting in a loss of incident solar energy. Therefore, some early-reported plasmonic OPVs doping nanoparticles into a hole/electron extraction film obtained limited increases in PCE with enhancement factors of universally <20%^17^,^18^.

Recently, some research groups have tried to wrap metal nanoparticles in a dielectric layer and then physically doped these core-shell structures into an active layer or inserted them at the interface of a carrier extraction film/an active layer^3^,^19^,^20^. For example, Chen *et al*. wrapped the gold nanospheres within a 50 nm silica shell and then located them into PEDOT:PSS in OPVs. The thick silica shell layer makes these nanospheres penetrate into poly(3-hexylthiophene):[6,6]-phenyl-C_61_-butyric acid methyl ester (P3HT:PC_61_BM) layer, which is just like a doping of gold nanoparticles into the active layer, generating an increase of 15.5% in efficiency^21^. Xu *et al*. synthesized the Au NR@SiO_2_ core-shell structures and doped them into P3HT:PC_61_BM and poly[2,6-(4,4-bis-(2-ethylhexyl)-4*H*-cyclopenta[2,1-b;3,4-b’] dithiophene)-*alt*-4,7-(2,1,3-benzothiadiazole)]:[6,6]-phenyl-C_71_-butyric acid methyl ester (PCPDTBT:PC_71_BM) cells, realizing 12.9% and 26% enhancement factors for PCE^22^. It is worth noting that they did not mention the thickness of the silica shell in their paper. In their following work, they fabricated a dual plasmonic OPV by respectively doping Au nanospheres and Au-silica nanorods into PEDOT:PSS and the 7,7′-(4,4-bis(2-ethylhexyl)-4*H*-silolo[3,2-*b*:4,5-*b*’]-dithiophene-2,6-diyl)bis(6-fluoro-4-(5′-hexyl-[2,2′-bithiophen]-5-yl)benzo[*c*][1,2,5]thiadiazole) (*p*-DTS(FBTTh_2_)_2_):PC_71_BM layer and obtained a PCE of 8.72% with a 31% enhancement over the control device^23^. It should be noted here the thickness of the silica shell layer is 7 nm in their surface plasmon-enhanced OPVs. Then they investigated thickness influences of silica shells on PCE and found a thinner shell of 5 nm is better than 7 and 10 nm ones, obtaining a 26% increase in PCE in the *p*-DTS(FBTTh_2_)_2_:PC_71_BM bulk heterojunction OPVs^24^. However, to our knowledge, nobody has further discussed the optimal shell thickness of metal SPs in organic/polymer solar cells. In Peng’s work^25^, he studied the distance dependence (the SiO_2_ thickness) of the energy transfer between a plasmon and a gain medium, and found that the direction of the energy transfer is dependent on the SiO_2_ thickness. Although he did not fabricate any device, his research work on the physical mechanism of the energy transfer indicates the SiO_2_ thickness plays an important role in energy transfer directions. So in our present paper, we chose Au NRs with a longitudinal/transverse ratio of ~2.0 and then wrapped these NRs in 3, 14 and 38 nm SiO_2_ shells. Note that a longitudinal/transverse ratio of ~2 generates a long/short axis resonance of 608/520 nm in aqueous solution and even if transferring these NRs into a solid film, the red shift for the longitudinal axis resonance is within 50–60 nm^12^, with these NR’s absorption still being located within the absorption range of the photoactive layer. By inserting these NRs at the interface of PEDOT:PSS and thieno[3,4-b]thiophene/benzodithiophene (PTB7):PC_71_BM, we observed different improvements on PCE and found the optimal performance occurs at an ultrathin SiO_2_ shell layer of ~3 nm with enhancement factors of 27–28% and 28–29% for PCE and *J*_sc_, realizing a 9.55% PCE and a 21.2 mA cm^−2^ *J*_sc_. To our knowledge, this is the best result using a single kind of metal nanoparticles in plasmonic OPVs.

## Results

**Figure 1:**
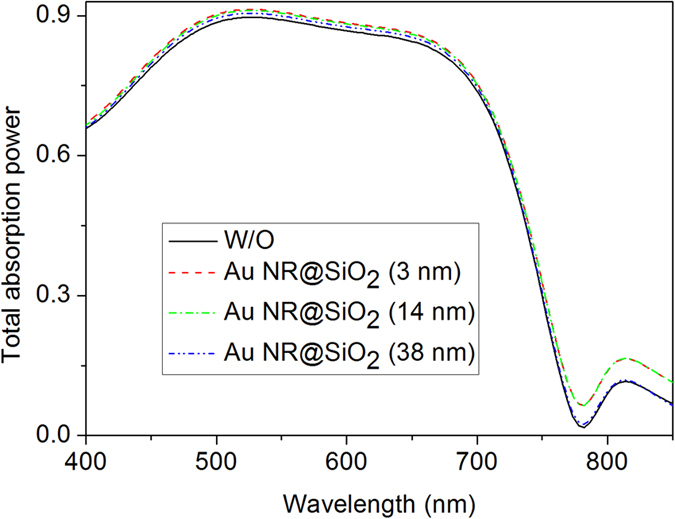
The simulated absorption power of the PTB7:PC_71_BM active layer with or without Au NRs@SiO_2_.

**Figure 2:**
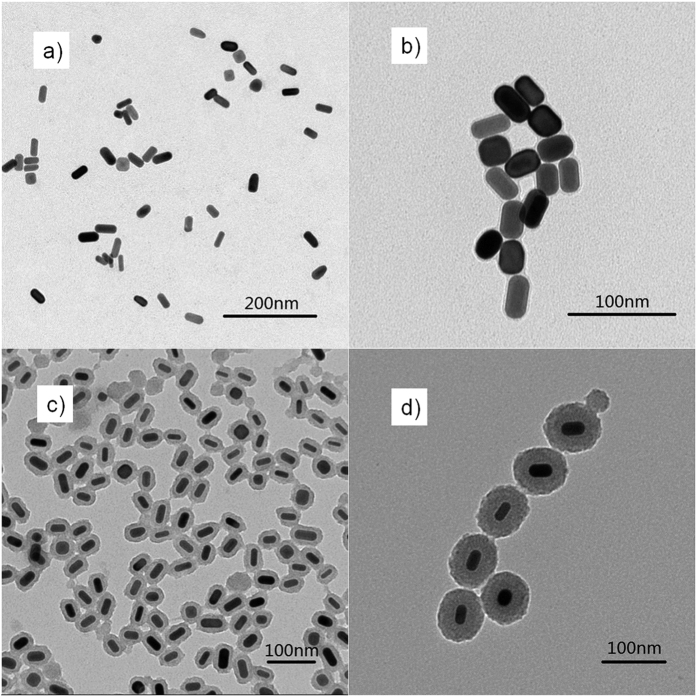
The TEM images for Au NRs coated with (**a**) 0, (**b**) 3, (**c**) 14, and (**d**) 38 nm silica shells.

**Figure 3:**
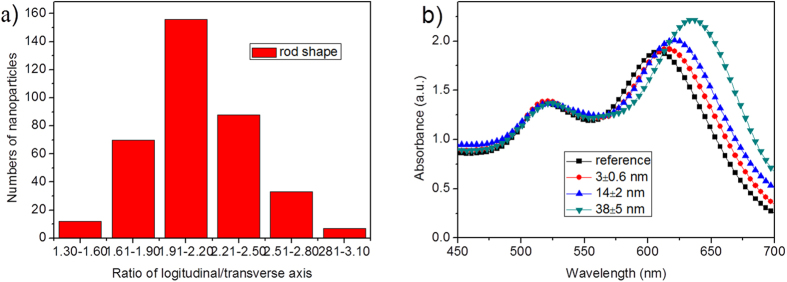
(**a**) The statistics data on the longitudinal/transverse ratios of as-synthesized Au NRs, and (**b**) the absorption spectra of the Au NRs with different silica shells.

Before synthesizing Au core-shell nanoparticles and manufacturing plasmonic OPVs, we first simulated the absorption power of the active layers by locating Au NR@SiO_2_ with alterable SiO_2_ shell thicknesses at the PEDOT:PSS/PTB7 interface. As the simulated film absorption shown in Fig. 1, we observed a more obvious absorption occurs in the PTB7:PC_71_BM layer with a thinner silica shell layer. It is especially significant at 750–850 nm with a peak of ~810 nm when the silica layer is no more than 14 nm. As Hsiao’s report^13^, the resonance wavelength near the cutoff wavelength of the PTB7 donor will be very beneficial for enhancing the light absorption ability of the photoactive layer. In order to verify this point and simultaneously observe the influence of the silica shell thickness on PCE, we synthesized Au NR core-shell nanostructures with different SiO_2_ thicknesses, e.g., 3, 14, and 38 nm, with their transmission electron microscope (TEM) images shown in Fig. 2. Analysis on ~380 Au NRs without silica coatings, we gave the detailed statistics data on the longitudinal/transverse ratios, as the histogram shown in Fig. 3a. We found that it mainly consists of rod shape nanoparticles with longitudinal/transverse ratios of 2.0 ± 0.5, among which ~43% particles own a longitudinal/transverse ratio of 2.0 ± 0.1, demonstrating a good homogeneity in our nanoparticles. These Au NRs were further wrapped with silica thicknesses of about 3 ± 0.6, 14 ± 2 and 38 ± 5 nm by adding different volumes of tetraethylorthosilicate (TEOS) into the final nanorods solutions. The as-synthesized Au NRs with 3 ± 0.6, 14 ± 2, and 38 ± 5 nm silica shells are shown in Fig. 2b–d, from which we find dense and uniform silica coatings on Au NRs. And these dense and uniform silica coatings sufficiently avoid exciton quenching through an efficient isolation of excitons from NRs. With the increase in silica coating thickness, the absorption for Au NR’s longitudinal axis exhibits an obvious red shift from 608 nm (without silica shell) to 635 nm (38 nm silica shell), as shown in Fig. 3b, and this trend is in accordance with previous reported results^26^. In contrast, the absorption of the transverse axis shows an ignorable alteration with the resonance wavelength mainly locating at ~520 nm (520, 522 and 524 nm for 3, 14 and 38 nm silica shells).

To fully use the magnified local field and light scattering induced by these gold surface plasmons, we’d better locate these metal nanoparticles into an active layer or at the interface of carrier extraction layer/photoactive layer. In our previous experiments, we found physically doping metal nanoparticles into a photoactive layer, especially in PTB7:PC_71_BM blend solution, brings some negative effects on the micromorphology of the photoactive film (e.g., phase separation)^27^, leading to a decline in PCE although these metal nanoparticles are covered with dielectric shells. So in the present work, we inserted Au NR@SiO_2_ core-shell nanostructures at the interface of PEDOT:PSS/PTB7:PC_71_BM to try to reduce the negative influence of these nanoparticles on the film morphology of the PTB7:PC_71_BM active layer. Although Au NRs@SiO_2_ were spincoated onto PEDOT:PSS before forming PTB7:PC_71_BM, they were almost totally surrounded by the following PTB7:PC_71_BM photoactive layer in our device structure, which indicates a large part of the plasmon-enhanced electric field can be absorbed by the photoactive layer.

**Figure 4:**
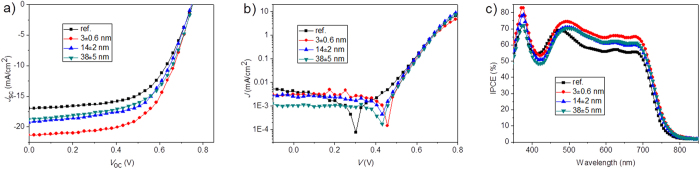
(**a**) *J-V* curves, (**b**) diode curves under dark conditions and (**c**) IPCE characteristics with and without Au NRs@SiO_2_ core-shell structures.

Through experimental exploration, we found the optimal distribution density for Au NRs@SiO_2_ is about one nanoparticle/2–3 μm^2^. We observed all plasmonic devices generate enhancements in PCE, among which the solar cell with 3 nm silica shell-coated Au NRs shows maximum enhancement factors of 27% and 28.5% for PCE and *J*_sc_, compared with the reference device without any nanoparticles (Fig. 4a and Table 1). This generates a PCE of as high as 9.55% (with a maximum value of 9.61%) in the OPV doped with Au NRs@3 nm SiO_2_, reaching a highest PCE level among reported plasmonic OPVs using a single kind of metal nanoparticles^28^,^29^. Increasing the SiO_2_ shell to 38 nm significantly declines PCE and *J*_sc_ to 8.25% and 18.5 mA cm^−2^, with only 9.3% and 12.1% enhancements. We summarized all parameters including *V*_oc_, *J*_sc_, FF, PCE, series resistance (*R*_s_) and shunt resistance (*R*_sh_) in Table 1, from which we noted that although doping a proper concentration of Au NRs brings a negligible effect on *V*_oc_ and FF, it greatly enhances *J*_sc_.

## Discussion

**Figure 5:**
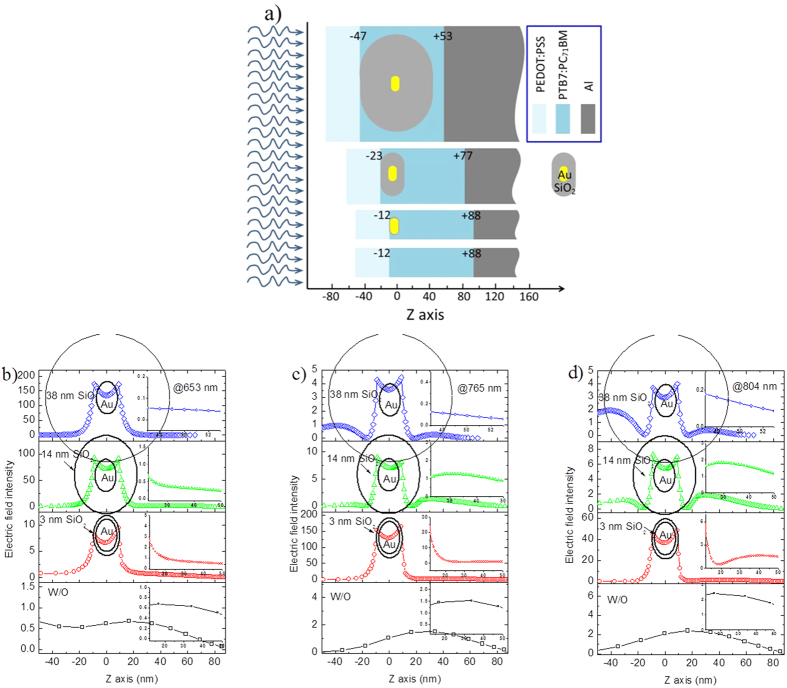
(**a**) The cross-section schematic of the plasmon-enhanced OPVs along Z axis (at Y = 0). Au NRs@SiO_2_ with 3, 14 and 38 nm silica shells are located in different coordinate positions in order to simulate the field distribution easily, where the PTB7:PC_71_BM active layer runs across the Z axis range of −12–88, −23–77 and −47–53 nm for doping Au NRs@SiO_2_ with 3, 14 and 38 nm silica shells. The PTB7:PC_71_BM layer without any Au NRs@SiO_2_ is located within −12–88 nm. The electric field distributions along with Z axis (both X = 0 and Y = 0) are calculated with FDTD software. (**b–d**) Respectively corresponds to 653, 765, and 804 nm and in each graph, it corresponds to the electric fields with 38, 14, 3 nm silica shell thicknesses from top to down. The bottom one is the electric fields without nanoparticles. Inset is the electric field intensity penetrated into the active layer.

**Figure 6:**
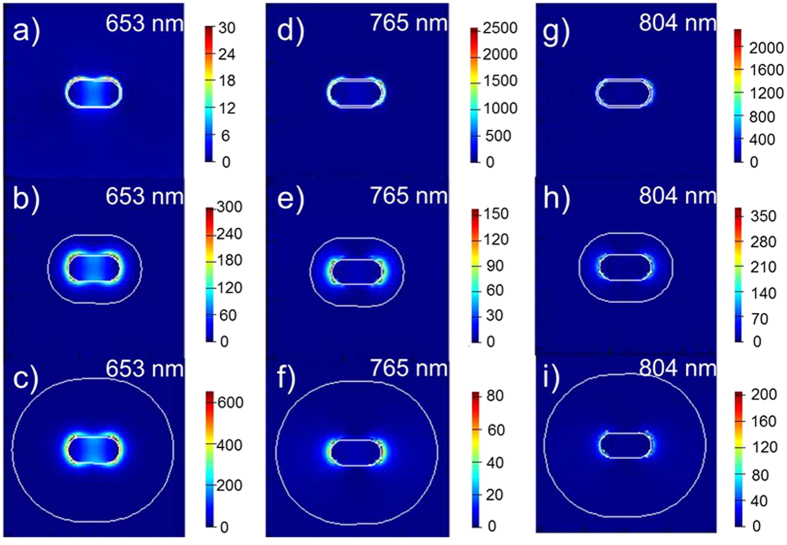
The local field distributions around Au NRs in plasmonic devices on XZ plane at Y = 0. Here, incident light propagation and polarized direction of this incident light beam are along Z and X axis, respectively. (**a,d,g**) Correspond to the fields at 653, 765, and 804 nm for Au NRs with 3 nm silica shells, (**b,e,h**) correspond to the fields at 653, 765, and 804 nm for Au NRs with 14 nm silica shells, while (**c,f,i**) correspond to the fields at 653, 765, and 804 nm for Au NRs with 38 nm silica shells.

**Figure 7:**
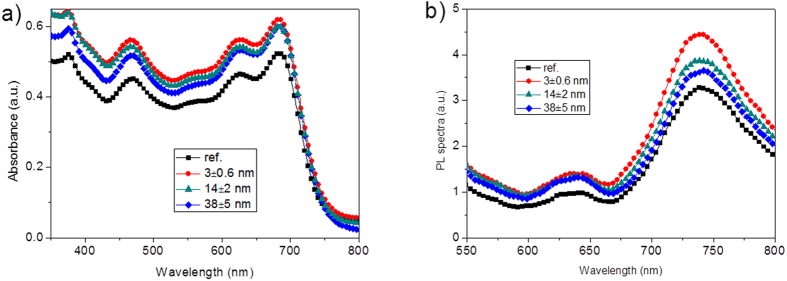
The measured (**a**) absorption and (**b**) PL spectra of the PEDOT:PSS/Au NRs@SiO_2_/PTB7:PC_71_BM films with 3, 14, and 38 nm silica shells. A standard structure of PEDOT:PSS/PTB7:PC_71_BM is also attached for comparison.

Significant increase in *J*_sc_, on one hand, is attributed to the scattering effect of Au NRs@SiO_2_ to the incident solar light, thus giving rise to a longer light path and further improving light absorption in OPVs^12^,^30^ and on the other hand, is due to an obviously enhanced local field induced by Au NR’s LSPR, promoting a further absorption of the active layer. Here, we calculated the electromagnetic field inside the cells in order to make sure of the role that the Au NRs played. It should be noted that only electric field intensity is considered due to a far larger intensity for the electric field than the magnetic one. Referring to the relative position of Au NRs in the solar cells in Fig. 5a (with Au NR’s longitudinal axis and light propagation direction respectively along X- and Z-axes), we compared the change of the electric field intensities with or without Au NRs@SiO_2_ at different wavelengths, with the simulation results shown in Fig. 5b–d, and drew some conclusions. Here, we supposed that the Au NRs distribute periodically along with XY plane and one of Au NRs runs across the center of our present coordinate system (this Au NR@SiO_2_’s center is located at 0 for X, Y, and Z axes). First, we found a maximum field in XZ plane at X = 0 occurs at 653 and 765 nm for 38 and 3 nm SiO_2_-coated Au NRs, generating maximum intensities of ~174 for Au NR@38 nm SiO_2_ and ~164 for Au NR@3 nm SiO_2_. Although a similar field magnification generated by Au NRs with 3 and 38 nm SiO_2_ shells, it degrades quickly in the thick SiO_2_-coated Au NRs and declines to 0.05 (at 653 nm) and 0.13 (at 765 nm) when reaching the PTB7 film, while it still remains high values of 2.6 (at 653 nm) and ~20 (at 765 nm) for 3 nm SiO_2_-covered ones. Above data indicate the Au NRs-induced large local field cannot be sufficiently used in the case with a thick silica shell and in contrast, the field remains a high level in the active layer when employing an ultrathin SiO_2_ shell. Thus, we should cover a shell as thin as possible onto metal nanoparticles to sufficiently utilize the Au-induced LSPR and 2–3 nm is the thinnest shell layer that we can control in our present experimental conditions. A shell of less than 2 nm will generate incomplete coverage, which leads to a significant exciton quenching on the surfaces of these Au NRs. Second, from the field distributions in XZ plane (at Y = 0) and along with Z axis shown in Figs 5 and 6, we mentioned that the local field induced by Au NRs is asymmetrical along with the propagation direction of the incident light (Z axis). For instance, the local field at the contact surface of Au NRs@SiO_2_ and PEDOT:PSS (Z = −47 nm in the top structure of Fig. 5a) is larger than that at the interface of Au NRs@SiO_2_ and PTB7 at Z = + 47 nm, which is especially obvious at 765 (Fig. 5c) and 804 nm (Fig. 5d, 804 nm is near the cut-off absorption wavelength of PTB7) for Au NRs with a 38 nm shell layer. Via promoting the PTB7 absorption near the bottom of nanoparticles at the PEDOT:PSS side, together with light scattering of Au NRs@SiO_2_, it generates a final 9.7% increase in PCE for the device with 38 nm SiO_2_-coated Au NRs. The measured absorption and PL spectra for the PEDOT:PSS/Au NRs@SiO_2_/PTB7:PC_71_BM multilayer in Fig. 7 is accordant with the above simulated results, with the 3 nm silica-coated Au NRs-inserted multilayer films showing a strongest light absorption ability and a highest PL intensity. Analysis on the simulated electric field distributions around Au NRs@SiO_2_ core-shell structures, we found the maximum value occurs at the nanorod’s shoulder (Fig. 6), instead of its sides discussed above, so we clearly observed the maximum LSPR intensity, generated by a thin silica shell layer of 3 nm, reaches ~2500, far larger than ~350 and ~600 for 14 and 38 nm silica shells. As a result, an extremely strong local field and a much slower attenuation of LSPR around 3-nm silica-coated Au NRs make the field intensity remain a high value even in the active layer, thus sufficiently improve the absorption of PTB7, accompanied with significant increases in *J*_sc_ and PCE.

In addition to the change of electromagnetic field inside OPVs, we also considered influences of Au NRs@SiO_2_ core-shell structures on the electrical transport properties of films. From Table 1, we found inserting Au NRs induces a slight decrease in *R*_s_, and it should be attributed to a beneficial influence of ethanol solvent instead of Au NRs^31^. In order to explain this problem, we investigated the influence of the ethanol solvent on OPV’s performances, where ethanol was used as Au NRs’ solvent in our plasmonic OPVs. We manufactured two groups of devices with a standard structure of ITO/PEDOT:PSS/PTB7:PC_71_BM/LiF/Al, and an ethanol-treated one with ethanol spincoated onto PEDOT:PSS (2000 rpm, same with metal nanoparticles’ rotation speed). The introduction of ethanol into the PEDOT:PSS/PTB7:PC_71_BM interface brings significant increases in FF and *J*_*s*c_, followed by a PCE increase, indicating a more efficient exciton dissociation in ethanol-treated device, as the summarized parameters (*J*_sc_, PCE, FF, *R*_s_ and *R*_sh_) in Figure S2 in Supplementary Information. The use of ethanol also results in a decline in *R*_s_. Thus, as a combination of positive and negative effects from ethanol and core-shell metal nanoparticles, *R*_s_ was finally observed to slightly decline in plasmonic OPVs, as shown in Table 1. In addition, *R*_sh_ was also observed with a slight increase and then a significant decline accompanied with the silica shell thickness. This means that a thicker silica shell onto metal nanoparticles on one hand restrains carrier transport due to a longer transport path for these dissociated excitons to overcome (from nanoparticle’s surface to the PEDOT:PSS layer), and on another hand leads to a thinner PTB7:PC_71_BM photoactive layer on these Au NRs, both of which make photon-generated excitons obviously decline with shell thickness’ increase. The calculated exciton generation rates in control device and plasmonic OPVs with 3, 14 and 38 nm silica thicknesses are 1.11 × 10^28^, 1.39 × 10^28^, 1.28 × 10^28^, and 1.26 × 10^28^ m^−3^ s^−1^ at saturation current densities of 177.5, 223.0, 204.6, and 202.3 A m^−2^, respectively. Detailed results are shown in Figure S3 in Supplementary Information. Above explanations also make it clear that a slight decline in the dark current density in plasmonic OPVs (Fig. 4b).

**Figure 8:**
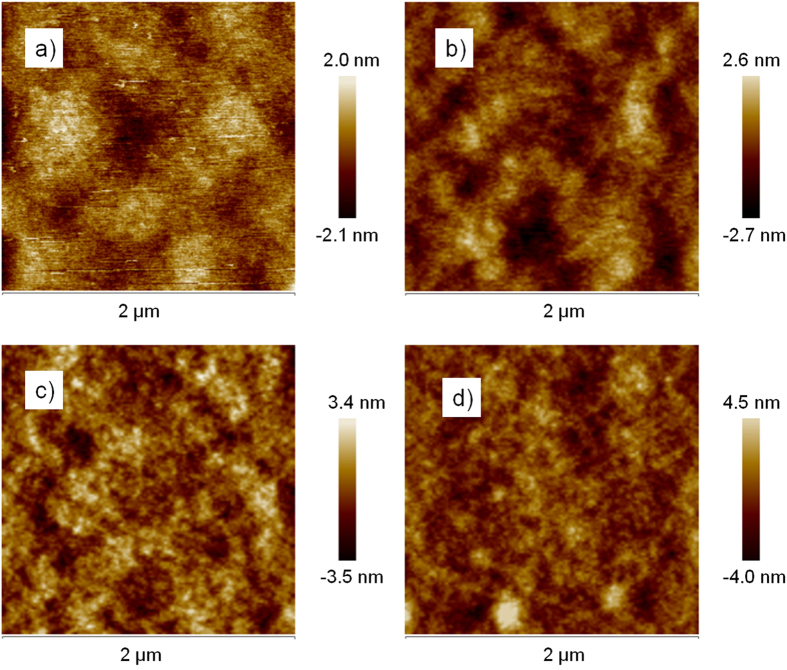
The surface morphology of the active layers with or without Au NRs@SiO_2_. (**a–d**) are in sequence the multilayers without metal nanoparticles (**a**) and with 3 (**b**), 14 (**c**) and 38 (**d**) nm silica shell on Au NRs.

Finally, the surface morphologies of the active layers with three kinds of Au NRs@SiO_2_ were measured by applying atomic force microscopy (AFM), where the PTB7:PC_71_BM film without metal nanoparticles was also measured for comparison, as can be seen in Fig. 8. The employment of Au NRs@SiO_2_ aroused some changes of the surface roughness (*R*_ms_) of the active layer, with a gradual increase in *R*_ms_ from 0.58 nm for the pure PTB7:PC_71_BM film to 0.76, 1.00, and 1.17 nm for the films inserted with Au NRs with 3, 14, and 38 nm SiO_2_ shells. The increase in surface roughness may enlarge the interface contact area of the PEDOT:PSS/active layer and the active layer/cathode, which is beneficial to exciton dissociation and electron extraction^8^,^11^, thus enhances *J*_sc_ and FF. However, constant values of FF in our measurement results demonstrate the insertion of Au NRs at the interface of PEDOT:PSS/active layer generates an ignorable influence on the exciton dissociation, suggesting a major mechanism on performance improvements is originated from absorption enhancement induced by SPs.

## Conclusions

As a result, we synthesized Au nanorods with different silica shell thicknesses and inserted them into the interface of PEDOT:PSS and PTB7:PC_71_BM. And we amazedly found the OPVs doped with Au NRs within a silica shell of as thin as 2–3 nm exhibit the highest *J*_sc_ and PCE. This is primarily due to an extremely strong local field generated by Au NRs with a thin silica shell. In addition, a much slower attenuation of LSPR around Au NRs@ 3 nm SiO_2_ makes the local field intensity remain a high value in the active layer, thus sufficiently improves the absorption of PTB7. We also explored the influence of the SiO_2_-coated Au NRs on film’s electrical property and found doping Au NRs@SiO_2_ slightly reduces the electrical conductivity ability of films. Our exploration work provides a clear design concept on precise control of core-shell metal nanoparticle’s shell thickness to realize high performances in plasmonic OPVs.

## Methods

### Materials

The following chemicals were purchased and used as received. Hexadecyltrimethylammonium bromide (CTAB, > 98.0%), tetrachloroaurate trihydrate (HAuCl_4_·3 H_2_O), sodium borohydride (NaBH_4_, 99%) and L-ascorbic acid (AA, >99.5% from BioUltra) were purchased from J&K Chemical. Sodium 3-methylsalicylate (>97.0%) were purchased from TCI America. Silver nitrate (AgNO_3_, >99%), tetraethylorthosilicate (TEOS, 99%), 1,8-diiodoctane and Al were purchased from Sigma Aldrich. PEDOT:PSS (AI 4083), PTB7, PC_71_BM and LiF were bought from Baytron P, 1-Material, Nano C, and Hanfeng Chemical, respectively. Ultrapure water was produced with a Milli-Q Integral 5 system and used in Au NR synthesis experiments. All glassware were cleaned with aqua regia, rinsed extensively with water, and dried in an oven before use.

### Synthesis of Au NRs

The seed solution for Au NRs was prepared as previously reported^32^. 5 mL of 0.5 mM HAuCl_4_ was mixed with 5 mL of 0.2 M CTAB solution. 0.6 mL of fresh 0.01 M NaBH_4_ was diluted to 1 mL with water and was then injected into above Au(III)—CTAB mixture solution under vigorous stirring (1200 rpm). The solution color changed from yellow to brownish-yellow, and the stirring was stopped after 2 min. The seed solution was aged at room temperature for 30 min before use.

To prepare the growth solution, 0.28 g of CTAB together with 0.05 g sodium 3-methylsalicylate were dissolved in 10 mL of warm water (60 °C) in a 50 mL Erlenmeyer flask. The solution was allowed to cool to 30 °C, when a 240 μl of 4 mM AgNO_3_ solution was added. The mixture was kept undisturbed at 30 °C for 15 min, after which 10 mL of 1 mM HAuCl_4_ solution was added. After 15 min of slow stirring (400 rpm), a 50 μl of 0.64 M AA was added, and the solution was vigorously stirred for 30 s until it became colorless. The growth solution with a CTAB concentration of about 0.05 M was used right after preparation. Finally, 32 μl of seed solution was injected into the growth solution. The resultant mixture was stirred for 30 s and left undisturbed at 30 °C for 12 h for Au NRs growth. The reaction products were further treated with a centrifugation of 8500 rpm for 25 min to remove the raw materials.

### Synthesis of Au NR@SiO_2_

To coat the Au NRs with SiO_2_, 0.1 mM of NaOH solution was added into 5 ml of Au NRs solution to adjust the pH value to 10–11. Then TEOS was added to the solution under gentle stirring and the reaction was allowed to proceed for approximately 12 h in 90 °C. Adding 3, 10 and 15 μl of TEOS to the nanorods solutions generated the silica shell thicknesses of about 3 ± 0.6, 14 ± 2 and 38 ± 5 nm, respectively. The Au@SiO_2_ core@shell nanostructures were finally obtained with a centrifugation of 8500 rpm for 15 min to remove the surfactant and then re-dispersed into 5 ml ethanol.

### Plasmonic OPV device fabrication

OPVs were fabricated on a 180 nm-thick indium tin oxide (ITO)-coated glass substrate as our previously reported procedure^28^. Pre-patterned ITO coated glass substrates (7 Ω/square) were cleaned in sequence with acetone, ethanol, and deionized water for 10 min, and then blown with a N_2_ gas. After a 100 °C heat treatment for 20 min, the substrates were treated with UV O_3_ for 7 min and then transferred onto a spincoater to form a ~40 nm PEDOT:PSS layer, followed by a dry process of 120 °C for 30 min. The ethanol solvent consisting of Au NR@SiO_2_ core@shell nanostructures were spin-coated onto the PEDOT:PSS film with different doping concentrations of nanoparticles to form an optimized distribution density. After that, the PTB7:PC_71_BM blend layer was spin-coated onto the PEDOT:PSS at 1100 rpm for 60 s, forming a film of ~100 nm. Note that PTB7 and PC_71_BM were mixed with a weight ratio of 10:15 mg in 1 ml chlorobenzene solvent and the additive 1,8-diiodoctane was then added into the mixture solution with a volume ratio of 3:97. The above blend solution was stirred for about 2 days before use. With a solvent drying for about 0.5 h, the active layer-coated samples were transferred into the vacuum chamber to thermally deposit a thin LiF and a thick Al cathode at 5 × 10^−4^ Pa, forming cells with the effective area of 0.1 cm^2^. The measurements on the current density-voltage (*J*-*V*) characteristics and the incident photon-to-electron conversion efficiency (IPCE) curves were operated with a Keithley 2400 sourcemeter under 100 mW cm^−2^ illumination (AM 1.5 G, Oriel Sol3A, 300 W) in an atmosphere environment and room temperature without further encapsulation.

## Additional Information

**How to cite this article**: Zhang, R. *et al*. Influence of SiO_2_ shell thickness on power conversion efficiency in plasmonic polymer solar cells with Au nanorod@SiO_2_ core-shell structures. *Sci. Rep*. **6**, 25036; doi: 10.1038/srep25036 (2016).

## Supplementary Material

Supplementary Information

## Figures and Tables

**Table 1:** Photovoltaic parameters for devices with and without Au NRs@SiO_2_.

SiO_2_ thickness (nm)	*V*_oc_ (V)	*J*_sc_ (mA/cm^2^)	FF	PCE (%)	*R*_s_ (Ω cm^2^)	*R*_sh_ (Ω cm^2^)
ref.	0.74	16.5	0.60	7.52	10.1	801
3 ± 0.6	0.74	21.2	0.60	9.55	7.4	923
14 ± 2	0.74	19.1	0.60	8.53	8.7	322
38 ± 5	0.74	18.5	0.60	8.25	7.8	435

## References

[b1] Blom P. W. M., Mihailetchi V. D., Koster L. J. A. & Markov D. E. Device physics of polymer: fullerene bulk heterojunction solar cells. Adv. Mater. 19, 1551–1566 (2007).

[b2] Brédasb B. & Kippelen J.-L. Organic photovoltaics. Energ. Environ. Sci. 2, 251–261 (2009).

[b3] Xie F. X., Choy W. C. H., Wang C. C. D., Sha W. E. I. & Fung D. D. S. Improving the efficiency of polymer solar cells by incorporating gold nanoparticles into all polymer layers. Appl. Phys. Lett. 99, 153304 (2011).

[b4] Gan Q. Q., Bartoli F. J. & Kafafi Z. H. Plasmonic-enhanced organic photovoltaics: breaking the 10% efficiency barrier. Adv. Mater. 25, 2385–2396 (2013). 23417974 10.1002/adma.201203323

[b5] Pandey A. K., Aljada M., Velusamy M., Burn P. L. & Meredith P. Nanostructured, active organic–metal junctions for highly efficient charge generation and extraction in polymer-fullerene solar cells. Adv. Mater. 24, 1055–1061 (2012). 22271224 10.1002/adma.201103896

[b6] Chen J.-D. . Enhanced light harvesting in organic solar cells featuring a biomimetic active layer and a self-cleaning antireflective coating. Adv. Energy Mater. 4, 1301777 (2014).

[b7] Chen J.-D. . Single-junction polymer solar cells exceeding 10% power conversion effi ciency. Adv. Mater. 27, 1035–1041 (2015). 25408480 10.1002/adma.201404535

[b8] Chen F. C. . Plasmonic-enhanced polymer PVs incorporating solution-processable metal nanoparticles. Appl. Phys. Lett. 95, 013305 (2009).

[b9] Kim S. S., Na S. I., Jo J., Kim D. Y. & Nah Y. C. Plasmon enhanced performance of organic solar cells using electrode posited Ag nanoparticles. Appl. Phys. Lett. 93, 073307 (2008).

[b10] Yao K. . A general route to enhance polymer solar cell performance using plasmonic nanoprisms. Adv. Energy Mater. 4, 1400206–1400212 (2014).

[b11] Lee J. H., Park J. H., Kim J. S., Lee D. Y. & Cho K. High efficiency polymer solar cells with wet deposited plasmonic gold nanodots. Org. Electron. 10, 416–420 (2009).

[b12] Zhang Y. P. . Plasmonic-enhanced polymer photovoltaic cells based on Au nanoparticles with wide absorption spectra of 300–1000 nm. J. Mater. Chem. C 2, 9303–9310 (2014).

[b13] Hsiao Y. S. . Improving the light trapping efficiency of plasmonic polymer solar cells through photon management. J. Phys. Chem. C 116, 20731–20737 (2012).

[b14] Ng A. . Enhanced performance of PTB7:PC_71_BM solar cells via different morphologies of gold nanoparticles. ACS Appl. Mat. Interfaces 6, 20676 (2014). 10.1021/am504250w 25408486

[b15] Baek S.-W. . Enhanced efficiency of solution-processed small molecule solar cells upon incorporation of gold nanospheres and nanorods into organic layers. ACS Nano 8, 3302–3312 (2014). 24652441 10.1039/c4cc01322k

[b16] Chen S. F. . Plasmon-enhanced polymer photovoltaic cells based on large aspect ratio gold nanorods and the related working mechanism. Appl. Phys. Lett. 104, 213903 (2014).

[b17] Jankovi V. . Active layer-Incorporated, spectrally tuned Au SiO_2_ core shell nanorod-based light trapping. ACS Nano 7, 3815–3822 (2013). 23627699 10.1021/nn400246q

[b18] Kim I., Jeong D. S., Lee T. S., Lee W. S. & Lee K.-S. Plasmonic absorption enhancement in organic solar cells by nano disks in a buffer layer. J. Appl. Phys. 111, 103121 (2012).

[b19] Wang D. . Enhancement of donor–acceptor polymer bulk heterojunction solar cell power conversion efficiencies by addition of Au nanoparticles. Angew. Chem. Int. Edit. 50, 5519–5523 (2011). 10.1002/anie.201101021 21520371

[b20] Li X. H., Choy W. C. H., Lu H. F., Sha W. E. I. & Ho A. H. P. Efficiency enhancement of organic solar cells by using shape-dependent broadband plasmonic absorption in metallic nanoparticles. Adv. Funct. Mater. 23, 2728–2735 (2013).

[b21] Chen B. X. . Surface plasmon enhancement of polymer solar cells by penetrating Au/SiO_2_ core/shell nanoparticles into all organic layers. Nano Energy 2, 906–915 (2013).

[b22] Xu X. Y. . A plasmonically enhanced polymer solar cell with gold–silica core–shell nanorods. Org. Electron. 14, 2360–2368 (2013).

[b23] Xu X. Y. . Enhanced efficiency of solution-processed small molecule solar cells upon incorporation of gold nanospheres and nanorods into organic layers. Chem. Commun. 50, 4451–4454 (2014). 10.1039/c4cc01322k 24652441

[b24] Xu X. Y. . Effect of shell thickness on small-molecule solar cells enhanced by dual plasmonic gold-silica nanorods. Appl. Phys. Lett. 105, 113306 (2014).

[b25] Peng B. . Fluorophore-doped core-multishell spherical plasmonic nanocavities: resonant energy transfer toward a loss compensation. ACS Nano 6, 6250–6259 (2012). 22690741 10.1021/nn301716q

[b26] Gangishetty M. K., Scott R. W. J. & Kelly T. L. Panchromatic enhancement of light-harvesting efficiency in dye sensitized solar cells using thermally annealed Au@SiO_2_ triangular nanoprisms. Langmuir 30, 14352–14359 (2014). 25369560 10.1021/la503878m

[b27] Xue M. . Charge-carrier dynamics in hybrid plasmonic organic solar cells with Ag nanoparticles. Appl. Phys. Lett. 98, 253302 (2011).

[b28] Li X. . Dual plasmonic nanostructures for high performance inverted OSC. Adv. Mater. 24, 3046–3052 (2012). 22566360 10.1002/adma.201200120

[b29] Lu L. Y., Luo Z. Q., Xu T. & Yu L. P. Cooperative plasmonic effect of Ag and Au nanoparticles on enhancing performance of polymer solar cells. Nano Lett. 13, 59–64 (2013). 23237567 10.1021/nl3034398

[b30] Hao J. Y. . Broadband plasmon-enhanced polymer solar cells with power conversion efficiency of 9.26% using mixed Au nanoparticles. Opt. Commun. 362, 50–58 (2016).

[b31] Tan Z. A. . High performance polymer solar cells with as-prepared zirconium acetylacetonate film as cathode buffer layer. Sci. Rep. 4, 4691–4699 (2014). 24732976 10.1038/srep04691 PMC3986729

[b32] Ye X. C. . Improved size-tunable synthesis of monodisperse gold nanorods through the use of aromatic additives. ACS Nano 6, 2804–2817 (2012). 22376005 10.1021/nn300315j

