# Supplementary material for: Influence of SiO2 shell thickness on power conversion efficiency in plasmonic polymer solar cells with Au nanorod@SiO2 core-shell structures
Source: Sci Rep. 2016 Apr 29;6:25036. doi: 10.1038/srep25036 (PMC4850401; doi:10.1038/srep25036)
Supplement: Supplementary Information [file srep25036-s1.pdf]

Supplementary Information

Influence of SiO_2 shell thickness on power conversion efficiency in plasmonic polymer solar cells with Au nanorod@SiO_2 core-shell structures

Ran Zhang[1], Yongfang Zhou[1], Ling Peng[1], Xue Li[2], Shufen Chen[1,3], Xiaomiao Feng[1], Yuqiao Guan[1] & Wei Huang[1,3]

[1]Key Laboratory for Organic Electronics and Information Displays & Institute of Advanced Materials (IAM), Jiangsu National Synergetic Innovation Center for Advanced Materials (SICAM), Nanjing University of Posts & Telecommunications (NUPT), Nanjing 210023, China.

[2]Mechanical Engineering Institute, Nanjing Institute of Technology, Nanjing 211167, China.

[3]Key Laboratory of Flexible Electronics (KLOFE) & Institute of Advanced Materials (IAM), Jiangsu National Synergetic Innovation Center for Advanced Materials (SICAM), Nanjing Tech University, 30 South Puzhu Road, Nanjing 211816, China.

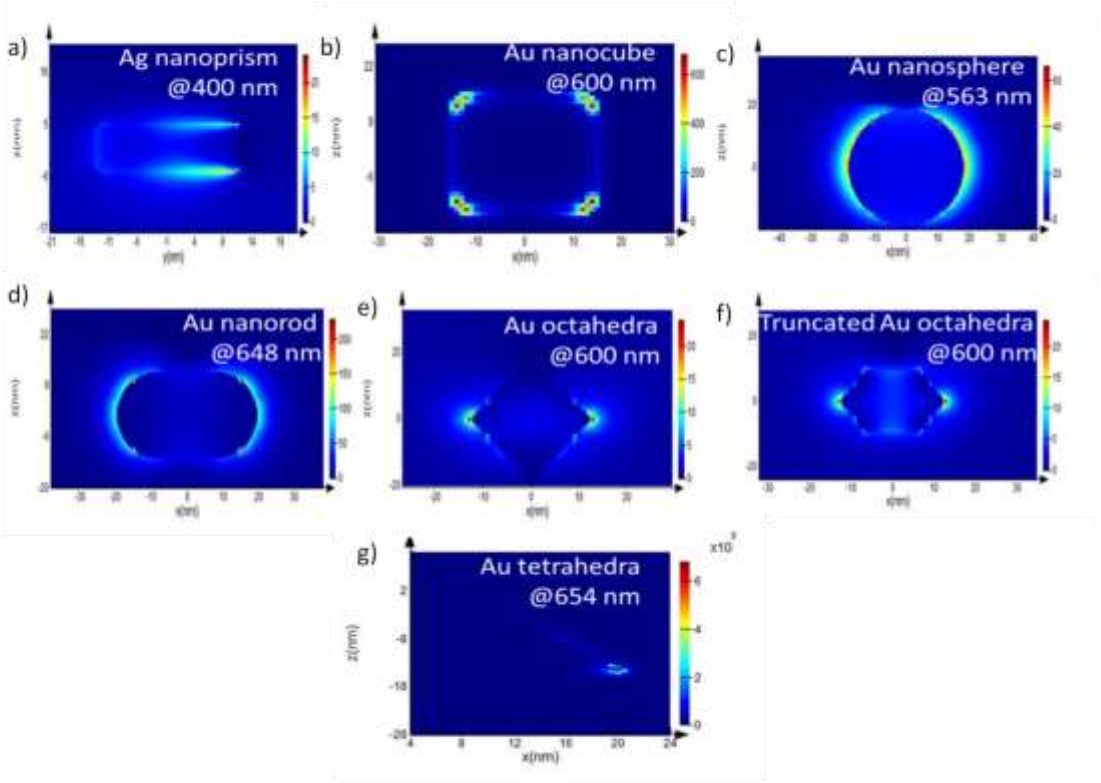

Figure S1. The local field distributions for different shapes of metal nanoparticles in plasmonic OPVs in XZ plane at Y=0. Here, incident light propagation and polarized direction of this incident light beam are along Z and X axis, respectively. The strongest localized field distributions at 400 nm for Ag nanoprisms (30 nm edge length and 10 nm thickness) (a), at 600 nm for Au nanocubes (28 nm side length) (b), at 563 nm for Au nanospheres (36 nm diameter) (c), at 648 nm for Au nanorods (39 and 23 nm for longitudinal and transverse axes, respectively) (d), at 600 nm for Au octahedras (36 nm diagonal length) (e), at 600 nm for truncated Au octahedras (18 nm distance for two parallel surfaces along Z axis and 36 nm diagonal length in XY plane) (f), and at 654 nm for Au tetrahedras (36 nm edge length) (g).

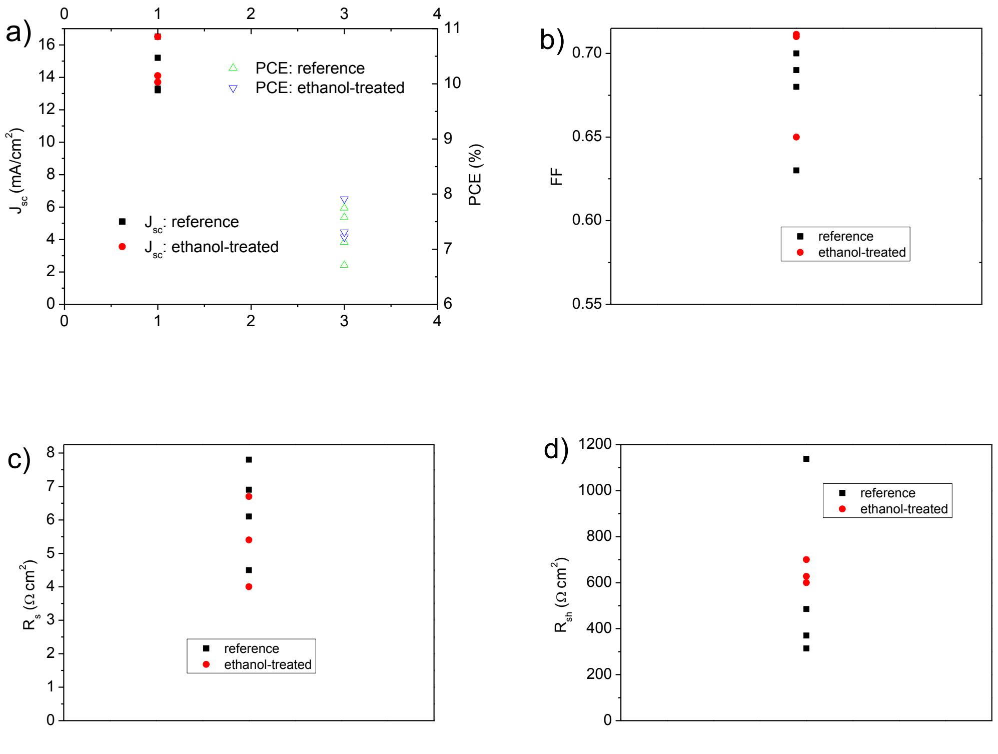

Figure S2. (a) J_{sc}, PCE, (b) FF, (c) R_s and (d) R_{sh} for ethanol-treated and standard OPVs.

<u>Calculation of exciton generation rates:</u>

To explore the effects of the silica thickness of Au NRs@SiO_2 on exciton generation, we calculated the maximum exciton generation rate (G_{max}) of our OPV devices. Figure S3 reveals the effect of LSPR on photocurrent density (J_{ph}) versus effective voltage (V_{eff}). J_{ph} is determined as $J_{ph}=J_L-J_D$, where J_L and J_D are the current density under illumination and in the dark, respectively. V_{eff} is determined as $V_{eff}=V_0-V_a$, where V_0 is the voltage at which J_{ph}=0 and V_a is the applied bias voltage. Figure S3 clearly shows that J_{ph} increases linearly at low V_{eff} range and saturates at a high V_{eff} (i.e., V_{eff}=1.0 V). Assuming that all the photo-generated excitons are dissociated into free charge carriers and collected by electrodes afterward at a high V_{eff} region, saturation current density (J_{sat}) is then only limited by total amount of absorbed incident photons. G_{max} could be calculated from $J_{ph}=qG_{max}L$, where q is the electronic charge and L is the thickness of active layer

(100 nm for our PTB7:$PC_{71}BM$ layer). The calculated values of G_{max} for the control device is 1.11 $\times 10^{28}$ m^{-3} s^{-1} (J_{sat}=177.5 A m^{-2}). While Au nanorods are inserted, the value increases. We find the highest G_{max} value occurs at an ultrathin SiO_2 shell layer of ~3 nm, which is 1.39 $\times 10^{28}$ m^{-3} s^{-1} (at J_{sat}=223.0 A m^{-2}). With the thicknesses increase to 14 and 38 nm, Gs_{max} reduce to 1.28 $\times 10^{28}$ m^{-3} s^{-1} (J_{sat}=204.6 A m^{-2}) and 1.26 $\times 10^{28}$ m^{-3} s^{-1} (J_{sat}=202.3 A m^{-2}). The incorporation of NRs@SiO_2 impressively enhances G_{max} and since G_{max} is related with the maximum absorption of incident photons, the increase in G_{max} suggests an increased light absorption in Au NRs@SiO_2 based OPVs.

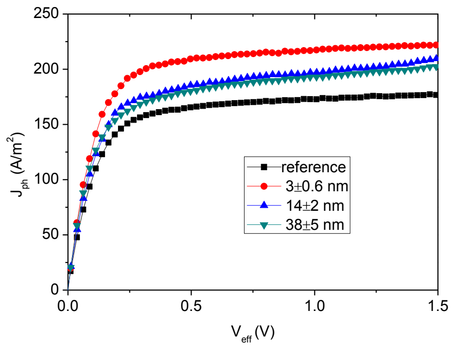

Figure S3. J_{ph}-V_{eff} characteristics of the control and plasmonic devices with 3, 14 and 38 nm silica thicknesses.
